# An algorithm for identifying causes of reoperations after orthopedic fracture surgery in health administrative data: a diagnostic accuracy study using the Danish National Patient Register

**DOI:** 10.2340/17453674.2024.42633

**Published:** 2025-01-13

**Authors:** Signe S JENSEN, Anders B RØNNEGAARD, Per H GUNDTOFT, Søren KOLD, Bjarke VIBERG

**Affiliations:** 1 Department of Orthopedic Surgery and Traumatology, Kolding Hospital; 2 Department of Clinical Research, University of Southern Denmark; 3 Department of Orthopedic Surgery and Traumatology, Aarhus University Hospital; 4 Department of Orthopedic Surgery, Aalborg University Hospital; 5 Institute of Regional Health Research, University of Southern Denmark; 6 Department of Orthopedic Surgery and Traumatology, Odense University Hospital, Denmark

## Abstract

**Background and purpose:**

Disease- or procedure-specific registers offer valuable information but are costly and often inaccurate regarding outcome measures. Alternatively, automatically collected data from administrative systems could be a solution, given their high completeness. Our primary aim was to validate a method for identifying secondary surgical procedures (reoperations) in the Danish National Patient Register (DNPR) within the first year following primary fracture surgery. The secondary aim was to evaluate the accuracy of the diagnosis and procedure codes used to determine the causes of these reoperations. Finally, we developed algorithms to enhance precision in identifying the reasons for reoperations.

**Methods:**

In a national cohort of 11,551 patients with primary fracture surgery, reoperations were identified through subsequent surgical procedure codes in the DNPR. Each patient record was reviewed to confirm the reoperations and causes. To improve accuracy, a stepwise algorithm was developed for each cause.

**Results:**

We identified 2,347 possible reoperations; 2,212 were validated as true reoperations by review of patient record, i.e., a 94% positive predictive value (PPV). However, the coding for the causes of these reoperations was inaccurate. Our algorithm identified major reoperations with a sensitivity/PPV of 89/77%, minor reoperations 99%/89%, infections 77/85%, nonunion 82/56%, early re-osteosynthesis 90/75%, and secondary arthroplasties 95/87%.

**Conclusion:**

While the overall reported reoperations in the DNPR had a high PPV, the predefined diagnosis and procedure codes alone were not sufficient to accurately determine the causes of these reoperations. An algorithm was developed for this purpose, yielding acceptable results for all causes except nonunion.

Surgical treatment of fractures is common and, despite treatment advancements, complications like poor bone healing, infection, pain, and diminished quality of life persist [1,2]. The relatively low incidence of nonunion and infection (5–10%) requires large, population-based datasets to conduct risk-factor analyses [3,4]. While some fracture registers, such as the American College of Surgeons National Surgical Quality Improvement Program (NSQIP), may provide useful information, they are insufficient for tracking reoperations. NSQIP monitors patients only for 30 days post-surgery, which is not adequate for detecting complications like nonunion [5]. Another example is the Swedish Fracture Register, which contains detailed surgeon-reported data on more than 1,000,000 fractures and provides real-time aggregated statistics. However, despite its extensive dataset, the completeness of reoperation data is 63%, suggesting that surgeon-reporting registries are insufficient regarding reoperations [6]. Because of external validity issues and limitations in data infrastructure, it is not always feasible to maintain high-quality registers.

As a substitute, it might be reasonable to evaluate health administrative databases. These databases could be particularly beneficial for assessing rare events and everyday surgical practices, due to their large data volumes, high completeness and coverage [7]. However, internal data validity is crucial to avoid misinterpretations and invalid conclusions. The Care Register for Health Care in Finland is an example of an otherwise complete and accurate administrative database. Nevertheless, studies show that subsidiary diagnoses and secondary procedures are poorly coded [8].

Our primary aim was to validate a method for identifying secondary surgical procedures (reoperations) in the Danish National Patient Register (DNPR) within the first year following primary fracture surgery. The secondary aim was to evaluate the accuracy of the diagnosis and procedure codes used to determine the causes of reoperations. Thirdly, we developed algorithms to enhance the precision in identifying the reasons for reoperations.

## Methods

### Study design, setting, and data access

We conducted a population-based diagnostic accuracy study in Denmark using routinely collected administrative discharge data. Reporting is performed in accordance with the Standards for Reporting of Diagnostic Accuracy (STARD), and the modified STARD criteria for health administrative data [9,10]. In Denmark, citizens are granted free and equal access to public hospital treatments in a tax-funded healthcare system, i.e., medical care is available to all. The allocation of the unique and personal central personal registration (CPR) number enables linkage from various registries in Denmark. The CPR number can only be altered when changing sex legally (occurred in 0.003% of the population at the time of our study) [11]. Public and private medical facilities are mandated to maintain comprehensive patient records, all organized around the CPR number [12,13]. The treatment of acute fractures is exclusively performed in the public healthcare system. Data was accessed through a secure connection to a remote desktop at the Danish Health Data Authorities. REDCap (Research Electronic Data Capture) electronic data capture tools hosted at OPEN Region of Southern Denmark was used for validation and enrichment. REDCap (https://project-redcap.org/) is a secure, web-based software platform designed to support data capture for research studies [14,15].

### Primary population

The study population is a national consecutive cohort of patients with fracture-related procedures, performed between January 1 and December 31, 2016. Patients were identified from the Danish Fracture Database (DFDB), which contains information on fracture type, treatment method, risk factors, and surgeon’s experience. DFDB is a surgeon-reporting database where surgeons enter data after procedures. In 2023, the database’s completeness was 55% (95% confidence interval [CI] 55–56), and the positive predictive value (PPV) for variables ranged from 81% to 100%, including 98% for operated side, surgery date, and surgery type [16]. For patients with multiple fractures, such as a wrist and hip fracture, each is treated as a separate observation. We excluded fractures of the spine, face, ribs, sternum, as well as primary arthroplasties.

### Reoperations

The CPR number enabled linkage between the DFDB and DNPR. The DNPR has documented person-specific information since 1995, and covers 99% of Danish hospital discharges [17]. Diagnosis and procedures are coded according to the International Classification of Diseases, version 10 (ICD-10) and Nordic Medico-Statistical Committee (NOMESCO), respectively [13,18]. Reoperations were defined as any surgical procedure code within the same anatomical area, related to the nervous system (KA), musculoskeletal system (KN), or skin (KQ) within the first 365 days of the primary procedure. As most patients have 2 extremities, we added a laterality criterion (side of the body affected). If the primary surgical procedure had a laterality diagnosis code (e.g., “right”), the secondary procedure must have been performed on the same side (e.g., “right”) or have missing laterality coding. We validated both major and minor reoperations. Major reoperations include procedures for infection and nonunion, early re-osteosynthesis within the first 6 weeks, and secondary arthroplasty that was not caused by nonunion or infection. Minor reoperations refer to procedures such as hardware removal and wound treatment, provided they are not related to an infection. When multiple reoperations occurred within the first year, we prioritized the most severe condition. The severity index was established in descending order: infection, nonunion, re-osteosynthesis, secondary arthroplasty, hardware removal, wound treatment, and others. Planned removal of K-wire or external fixation, and procedures for chronic foot ulcers, decubitus, and venous disease were not defined as reoperations and therefore excluded.

### Data collection and gold standard

Medical records were reviewed for each observation. The review team included main authors, physicians, medical students, and secretaries from respective hospitals. Key information included the indication for the secondary procedure, procedure and diagnosis codes, and risk factors. Personnel were trained to ensure accurate and unambiguous data registration. To minimize misclassification and interobserver bias, we established a predefined reoperation subgrouping based on time-to-surgery, plus diagnosis and procedure codes. Data collectors agreed or disagreed with the predefined reason for reoperation. If there was disagreement or missing data, the main author reviewed the entire surgical note for revalidation. Our gold standard was patients’ medical records where the following conditions were confirmed if the orthopedic surgeon mentioned them and took corresponding actions: infection, nonunion, early re-osteosynthesis, secondary arthroplasty, wound complications, and hardware removal.

### Statistics

**Figure 1:**
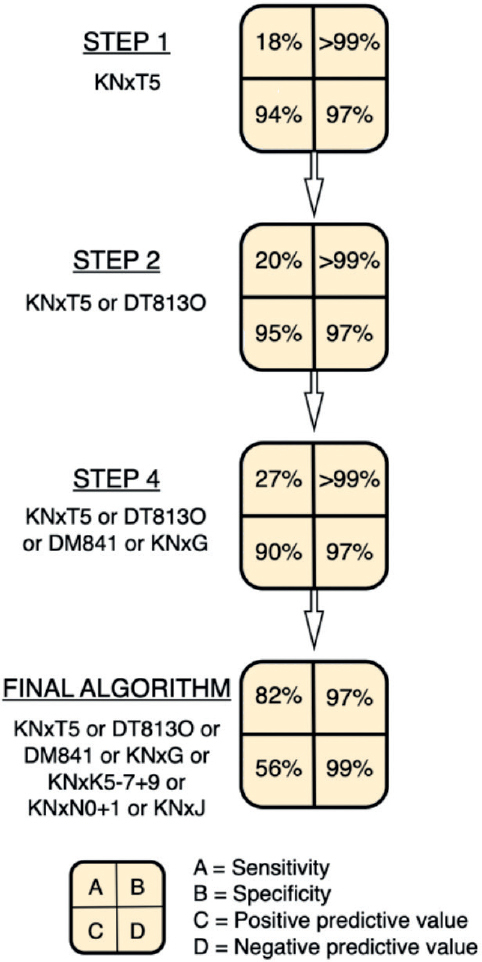
Sensitivity, specificity, and predictive values at each step. With each step, more codes are added to the previous ones using an “or” condition, which increases sensitivity but lowers the positive predictive value. This is a simplified version of the development of the algorithm to detect nonunion, which can be found in Supplementary materials. The design of this figure was adapted with permission from a colleague, Lars Lykke Hermansen.

We use the term “algorithm” to describe the collection of codes and timeframes used to identify reasons for reoperations with a certain level of accuracy [19]. We designed algorithms to identify the specific reasons for reoperations (infection, nonunion, etc.). The algorithms were built step-by-step, analyzing commonly used diagnosis and procedure codes for each cause of reoperation. We added codes using an “or” condition, meaning any of the listed codes would trigger the identification, rather than requiring all codes to be present (“and”) (Figure 1). Using logistic regression, we determined the best code matches, which helped increase sensitivity while maintaining an acceptable positive predictive value at each step. For both major and minor reoperations, we selected the steps with the highest kappa value for each cause of reoperation and combined them into 2 separate algorithms. We report the sensitivity, positive predictive value (PPV), Cohen’s kappa value, and area under the curve (AUC) for each algorithm used to identify the cause of reoperation as recommended by Ehrenstein et al. [19]. Specificity, negative predictive value, and ROC curves can be found in Supplementary data. Sensitivity was calculated as (true positives x 100)/(true positives + false negatives) and PPV was calculated as (true positives x 100)/(true positives + false positives). To determine the PPV for each cause of reoperation in the DNPR, we compared the recorded reason for reoperation in the DNPR with the reason documented by the surgeon in the medical record. However, the general identification of reoperations, and not the specific causes, is only presented with a positive predictive value. There is no sensitivity analysis as we do not know the false negatives in our primary cohort. We hypothesize that this number is small, since we included all additional surgical contacts within the first year in the same anatomical area.

The 95% confidence intervals (CI) are calculated using the exact Clopper–Pearson method. We analyzed data using Stata 17 (2021. Stata Statistical Software: Release 17; StataCorp LLC, College Station, TX, USA).

### Revalidation and missing data

We did not revalidate each individual site for data entry quality. However, we manually revalidated data on 625 patients. These patients were either marked with an incorrect assumed reason for reoperation or lacked a tick in the checkbox denoting the correct reason for reoperation. We found that data collectors had misclassified 23% of these patients. Patients were withdrawn from the final analysis in the case of missing data; for example we did not have medical records on patients reoperated on at private hospitals. Patients dying before the 1-year follow-up were not excluded.

### Ethics, registration, data sharing, funding, and disclosures

Ethics approval is not required for registry-based studies in Denmark. The Danish Patient Safety Authority approved the project (case number 3-3013-2729). Data sharing is not possible due to the regulations in the DNPR. However, we can be helpful with additional sub-analysis. The first author received grants from the following institutions: the Fond of Direktør Emil C. Hertz and Hustru Inger Hertz, Sygehus Lillebaelt, the University of Southern Denmark, the Region of Southern Denmark, and the Department of Orthopedics and Traumatology, Kolding Hospital. The funding did not influence interpretation of data, reporting of results, writing of the manuscript, or decision to publish. There are no conflicts of interest, and the decision to publish was made solely by the authors. Complete disclosure of interest forms according to ICMJE are available on the article page, doi: 10.2340/17453674.2024.42633

## Results

### Participants

**Figure 2:**
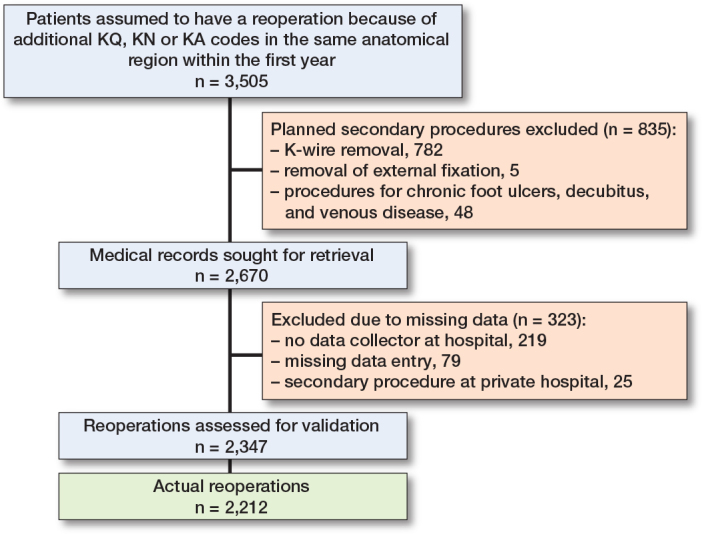
Flowchart overview of reoperations validated in the study.

11,874 primary osteosynthesis-related fracture procedures were registered in the DFDB and DNPR in 2016. Of the identified patients, 9% either died before a planned reoperation or before the 1-year follow-up. Vital status was unavailable for 35 patients. 60% did not have additional surgical procedures within 365 days, and 1,171 had surgical procedures in a different anatomical region. There were 139 procedures on the contralateral side. Therefore, we included 2,670 reoperations for validation (Figure 2). Medical records were unavailable for 323 reoperations, resulting in a cohort of 11,551 primary procedures and 2,347 reoperations. The reoperation cohort included 1,260 women (54%), with a median age of 52 years (interquartile age range 19–68). When we delved into anatomical specifics, the knee and lower leg (KNG category) accounted for 33% of all reoperations. Reoperation rate relative to primary surgery sites ranged from 9% to 33% (Table 1).

**Table 1:** Anatomical distribution of reoperations following osteosynthesis. Values are frequency of reoperations and percentage relative to primary surgery

Region	n (%)
Knee and lower leg (KNG)	300 (33)
Ankle and foot (KNH)	540 (32)
Elbow and forearm (KNC)	782 (23)
Pelvis (KNE)	9 (12)
Wrist and hand (KND)	124 (14)
Shoulder and upper arm (KNB)	153 (14)
Hip joint and thigh (KNF)	304 (8.9)

### Overall reoperations

We validated 2,347 expected reoperations, and 2,212 were confirmed as actual reoperations, resulting in a PPV of 94%. The remaining 135 cases were validated as: 104 emergency room visits, 13 chronic wound treatments, and 18 new fractures. Hardware removal was the predominant cause for reoperation, with a 1-year cumulative incidence of 7.3% (Table 2). The algorithms for each cause of reoperation are presented with the best fit between sensitivity, PPV, and kappa value in Table 3.

**Table 2:** Validated reasons for reoperations. Values are frequency and cumulative incidence during the 1-year follow-up period of the population at risk

Cause	n (%)
No reoperation	9,339 (81)
Hardware removal	870 (7.5)
Wound treatment	624 (5.4)
Re-osteosynthesis	195 (1.7)
Other	135 (1.2)
Infection treatment	114 (1.0)
Secondary arthroplasty	94 (0.8)
Nonunion treatment	94 (0.8)
K-wire removal	74 (0.6)
Removal of external fixation	12 (0.1)

**Table 3:** Algorithms to identify reasons for reoperations. Values with 95% confidence interval

Cause of reoperation	Sensitivity	PPV	K	AUC	TP	TrP	TN	TrN
All reoperations		94			2,347	2,212	9,204	
Major reoperations	89 (86–91)	77 (74–81)	0.64	0.91	570	441	1,777	1,721
Infection treatment	77 (68–85)	85 (77–92)	0.80	0.88	103	88	2,244	2,218
Nonunion treatment	82 (72–89)	56 (47–64)	0.65	0.90	138	77	2,209	2,192
Re-osteosynthesis	90 (85–94)	75 (69–81)	0.80	0.94	233	175	2,114	2,094
Secondary arthroplasty	95 (88–98)	87 (78–92)	0.90	0.97	104	90	2,234	2,238
Minor reoperations	99 (99–100)	89 (88–91)	0.82	0.92	1,667	1,485	680	671
Hardware removal	99 (98–100)	94 (92–96)	0.90	0.98	917	862	1,430	1,422
Wound treatment	99.5 (99–100)	83 (80–85)	0.86	0.96	753	621	1,594	1,591

Sensitivity and positive predictive value (PPV) for each algorithm. TP: test positive, TrP: true positives, TN: test negative, TrN: true negative. Sensitivity and positive predictive value are % with (95% confidence interval). K: Cohen’s kappa; AUC: area under the curve.

### Causes of reoperations

#### Major reoperations

Major reoperations included surgical treatment for infection, nonunion, re-osteosynthesis, and secondary arthroplasties. Our algorithm correctly identified 441 out of 497 patients, achieving a sensitivity of 89% (CI 86–91) and a PPV of 77% (CI 74–81). The algorithm used steps with the highest kappa value from various models (Supplementary Tables 1 to 7).

#### Minor reoperations

Minor reoperations included hardware removal and wound treatment. Our algorithm identified 1,485 out of 1,494 patients, with a sensitivity of 99% (CI 99–100), and PPV of 89% (CI 88–91).

#### Infection

114 patients had infections, which led to a 1-year cumulative incidence of 1% (Table 2). The correct procedure codes for infections (KNxW69 or KNxW59) were used in 30 patients (26%), while the correct diagnosis code (DT846) was used in 38 patients (33%). Only 11 patients (10%) had both the correct diagnosis and procedure codes, while 50% had either one or the other. The algorithm demonstrated a sensitivity of 77% (CI 68–85), and PPV of 85% (CI 7–92) (Supplementary Table 1, step 9).

#### Nonunion

The 1-year cumulative incidence of nonunion was 1%, including both diaphyseal and metaphyseal fractures (94 patients). The correct procedure code (KNxT5) was used in 17 patients (18%), while the correct diagnosis codes (DT813O or DM841) were used in 10 patients (11%). Only 7 patients (8%) had both the correct diagnosis and procedure codes, while 22% had either one or the other. The algorithm demonstrated a sensitivity of 82% (CI 72–89), and PPV of 56% (CI 47–64) (Supplementary Table 3, step 7).

#### Early re-osteosynthesis

195 patients had either re-osteosynthesis or secondary arthroplasties within the first 6 weeks, i.e., a 1-year cumulative incidence of 2%. The codes DT840–DT844, “mechanical complications related to orthopedic procedures”, were used in 14 patients (7%) only. The algorithm demonstrated a sensitivity of 90% (CI 85–94), and PPV of 75% (CI 69–81) (Supplementary Table 5, step 3).

#### Secondary arthroplasties

94 patients had secondary arthroplasties after 6 weeks from the primary surgery, resulting in a 1-year cumulative incidence of 1%. The correct procedure code (KNxC) was used in 3 patients (3%). The algorithm demonstrated a sensitivity of 95% (CI 88–98), and PPV of 87% (CI 78–92) (Supplementary Table 7, step 2).

#### Hardware removal

870 patients had hardware removal at least 6 weeks after the primary procedure. The correct procedure code (KNxU) was used in 862 patients (99%), with a sensitivity of 99% (CI 98–100) and a PPV of 94% (CI 92–96).

#### Wound complications

624 patients had a wound complication, defined as any surgical treatment involving KQ codes that were unrelated to infection. The correct procedure code (KQ) was used in 621 patients (99%), demonstrating a sensitivity of 99.5% (CI 99–100) and PPV of 83% (CI 80–85).

## Discussion

We conducted a nationwide validation study on reoperation codes following primary osteosynthesis, finding that overall reoperations can be identified with a PPV of 94% using data from the DNPR. We demonstrated that it is possible to develop algorithms that can be applied to data not primarily collected for research purposes, with good precision. This proof-of-concept could serve as an inspiration for other researchers who wish to implement similar methods in other fields. However, external validation should be performed to test the algorithm’s ability to perform in other environments and settings.

Administrative registers have clear limitations when used to identify causes of reoperations. When relying solely on explicit procedure or diagnosis codes, the sensitivity for identifying infections or nonunion is low and might lead to a major underestimation. Incorporating additional infection codes improved sensitivity to an acceptable level without reducing the PPV. However, this strategy is less effective for nonunion algorithms, where adding more codes lowered the PPV to a level only marginally above random selection.

Selecting an algorithm with low sensitivity but high PPV increases the likelihood that identified cases genuinely exhibit the condition, though this approach reduces the sensitivity, resulting in an incomplete capture of all cases. Alternatively, if the goal is to ensure comprehensive inclusion and manual record review is feasible, a high-sensitivity algorithm may be more appropriate, though with a lower PPV. Given that there is no standard definition of acceptable sensitivity and specificity, it ultimately depends on the trade-off between missing true positives and incorrectly classifying true negatives in studies. Therefore, the utility of an algorithm should be evaluated within the context of the specific study goals and the resources available.

A comparable study built a similar algorithm to identify dislocation cases after primary total hip arthroplasty and found a low sensitivity of 63% when both the expected and most specific diagnosis and procedure codes were required [20]. Following a similar approach, we avoided requiring both diagnosis and procedure codes for specific complications to be present.

A recent study revealed significant gaps in the DFDB, reporting an overall completeness of just 55% [16]. The database was terminated on March 30, 2020, as it was a surgeon-reporting database that did not merge information with the DNPR. Our data revealed a 1-year cumulative infection incidence of 1.0%, which aligns with a 2021 study that reported infection rates of 0.6–0.8% in patients with surgically treated hip fractures, specifically those treated with intramedullary nailing or sliding hip screws [21]. Therefore, we still believe that even with the incompleteness of the DFDB, our cohort remains representative of orthopedic procedures. Regarding our gold standard, we adhered strictly to what was documented and acted upon in the medical records. Future studies might benefit from further defining reoperations, e.g., by using standardized criteria such as proposed for infection [22]. An alternative strategy to identify reoperations could involve cross-checking data with secondary registers [23]. By monitoring and reporting reoperations, we might potentially improve the quality of care, even at the hospital level. Several registries have shown that reoperation rates can be reduced through such efforts. For example, in the Danish Multidisciplinary Hip Fracture Registry, the reoperation rate for non-displaced femoral neck fractures has dropped from 15% to around 8% [24]. In Norway, fewer uncemented stems are being used, and in Sweden, they have changed surgical approaches to avoid dislocations [25, 26]. These changes are likely due to several factors, with ongoing monitoring in national registries potentially being a key contributor to the improvements.

### Limitations

We solely evaluated cases provided they had a KN, KQ, or KA procedure code within the first year. The PPV of reoperations may have been overestimated, as we did not sample a cohort with negative cases in the primary cohort. The broad scope of the study, in which we examine all causes of reoperations within a year to determine the PPV of our identification method, also results in a very low actual proportion of nonunions and infections. This reduces the available data for these specific reoperations and could potentially lead to less precise PPV values. A 1-year follow-up may also be too short to accurately diagnose nonunion, so this study probably underestimates its true incidence. However, it is likely that the coding remains consistent over a longer follow-up period, though we do not have data to confirm this. We validated only the reoperation code and did not account for nonunions later found to be infected. Since microbiology results are unknown at the time of reoperation, some nonunions may have been infected. Incorporating this into algorithms is difficult because infections are not always recorded afterwards. Although this does not change the fact that the fracture has not healed, it becomes relevant when analyzing risk factors.

The primary patient cohort could be susceptible to selection bias, as we solely included patients registered in the DFDB while seeking data from the DNPR. Additionally, since we are collecting data on primary procedures, if surgeons are more likely not to report difficult surgeries, then the missing data (which pertains to potential reoperations) is related to the observed data (primary procedures). The likelihood of reoperations being missed therefore might depend on the difficulty of the primary procedure, which falls under the missing at random category.

### Conclusion

The developed algorithm was able to identify reoperations with a PPV of 94% and acceptable accuracy in identifying infections, early re-osteosynthesis, secondary arthroplasties, and both major and minor reoperations, though they remain less effective for nonunion.

*In perspective,* administrative databases offer extensive data, but they often lack detailed information. These registers are typically designed for settlement systems, insurance purposes, and monitoring specific diseases, not for research. Therefore, validating these databases according to specific research questions is crucial to avoid misinterpretation and development of algorithms may be an advantage for a more effective way of gaining new knowledge.

### Supplementary data

Supplementary Tables 1–8 and Supplementary Figures 1–4 are available as Supplementary data on the article page, doi: 10.2340/17453674.2024.42633

## Supplementary Material



## Notes

SSJ, ABR, BV, SK and PHG were responsible for the conception of the study. SSJ and ABR were responsible for data cleaning and analysis. SSJ drafted the manuscript. ABR, BV, SK, and PHG revised the manuscript.

Handling co-editors: Cecilia Rogmark and Philippe Wagner

*Acta* thanks Peter H J Cnudde and other anonymous reviewers for help with peer review of this manuscript.
