# Supplementary material for: An algorithm for identifying causes of reoperations after orthopedic fracture surgery in health administrative data: a diagnostic accuracy study using the Danish National Patient Register
Source: Acta Orthop. 2025 Jan 13;96:66–72. doi: 10.2340/17453674.2024.42633 (PMC11726852; doi:10.2340/17453674.2024.42633)
Supplement: Supplementary file 1 [file ActaO-96-42633-s1.pdf]

Supplementary material

Table 1: Steps in the infection algorithm

Number of infections	114
Number of reoperations without infections	2233

Step	Codes	Sensitivity	Specificity	PPV	NPV	TP	TrP	TN	TrN
1	KNxW69 (Reoperation for deep infection)	21.9 (14.7-30.6)	100 (99.8-100)	100 (86.3-100)	96.2 (95.3-96.9)	25	25	2322	2233
2	St1 + KNxW59 (Reoperation for superficial infection)	26.3 (18.5-35.4)	100 (99.8-100)	96.8 (83.3-99.9)	96.4 (95.5-97.1)	31	30	2316	2232
3	St2 + DT846 (Infection or inflammation due to internal fixation device)	50.0 (40.5-59.5)	99.7 (99.4-99.9)	90.5 (80.4-96.4)	97.5 (96.8-98.1)	63	57	2284	2227
4	St3 + DT847 (Infection and inflammatory reaction due to other internal orthopaedic device)	50.0 (40.5-59.5)	99.6 (99.4-99.9)	89.1 (78.8-95.5)	97.5 (96.8-98.1)	64	57	2283	2226
5	St4 + DT857 (Infection or inflammatory reaction due to other internal device)	50.9 (41.3-60.4)	99.7 (99.4-99.9)	89.2 (79.1-95.6)	97.5 (96.8-98.1)	65	58	2282	2226
6	St5 + DT814 (Infection following a procedure)	67.5 (58.1-76.0)	99.5 (99.1-99.7)	86.5 (77.6-92.8)	98.4 (97.7-98.8)	89	77	2258	2221
7	St6 + DM00 (Arthritis due to infectious agents and purulent arthritis)	68.4 (59.1-76-8)	99.5 (99.1-99.7)	86.7 (77.9-92.9)	98.4 (97.8-98.9)	90	78	2257	2221
8	St7 + DL02 (Abscess, funucle etc.)	70.2 (60.9-78.4)	99.3 (98.9-99.6)	84.2 (75.3-90.9)	98.5 (97.9-99.0)	95	80	2252	2218
9	St8 + KNxS (Incision and debridement of infection)	77.2 (68.4-84.5)	99.3 (98.9-99.6)	85.4 (77.1-91.6)	98.8 (98.3-99.2)	103	88	2244	2218
10	St9 + KNxU49* (Removal of internal fixation device)	92.1 (85.5-96.3)	95.7 (94.7-96.5)	52.0 (44.9-59.0)	99.6 (99.2-99.8)	202	105	2145	2136

* A maximum of 42 reoperation days. Sensitivity, specificity, positive predictive value (PPV), and negative predictive value (NPV) for each step of the algorithm identifying infections. TP: Test positive, TrP: True positives, TN: test negative, TrN: True negative. Values are % with (95% confidence interval).

Table 2: Data from the different steps of the infection algorithm

Step	Accuracy	Recall	Precision	False positive rate	Kappa	AUC
1	0.96	0.22	1.00	0.00	0.35	0.61
2	0.96	0.26	0.97	0.00	0.40	0.63
3	0.97	0.50	0.90	0.00	0.63	0.75
4	0.97	0.50	0.89	0.00	0.63	0.75
5	0.97	0.51	0.89	0.00	0.64	0.75
6	0.98	0.68	0.87	0.01	0.75	0.84
7	0.98	0.68	0.87	0.01	0.75	0.84
8	0.98	0.70	0.84	0.01	0.76	0.85
9	0.98	0.77	0.85	0.01	0.80	0.88
10	0.95	0.92	0.52	0.04	0.64	0.95

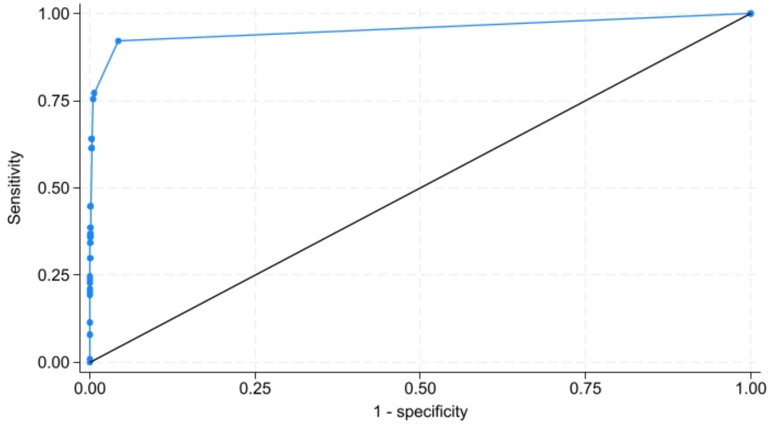

Figure 1: ROC-curve for infection algorithm

Table 3: Steps in the nonunion algorithm

Number of nonunions	94
Number of reoperations without nonunion	2253

Step	Codes	Sensitivity	Specificity	PPV	NPV	TP	TrP	TN	TrN
1	KNxT5 (Pseudoarthrosis surgery)	18.1 (11.0-27.6)	100 (99.8-100.0)	94.4 (72.7-99.9)	96.7 (95.9-97.4)	18	17	2329	2252
2	St1 + DT813O (Fracture slippage or pseudoarthrosis after reduction with or without fixation)	20.2 (12.6-29.8)	100 (99.8-100.0)	95.0 (75.1-99.9)	96.8 (96.0-97.5)	20	19	2327	2252
3	St2 + DM841 (Pseudoarthrosis)	22.3 (14.4-32.1)	100 (99.8-100.0)	95.5 (77.2-99.9)	96.9 (96.1-97.5)	22	21	2325	2252
4	St3 + KNxG (Excision, reconstruction and fusion)	27.1 (18.9-37.8)	99.7 (99.6-100)	89.7 (72.6-97.8)	97.1 (96.3-97.7)	29	26	2318	2250
5	St4 + KNxK5-7 + 9 (Osteotomies)	39.4 (29.4-50.0)	99.8 (99.5-100)	90.2 (76.9-97.3)	97.5 (96.8-98.1)	41	37	2306	2249
6	St5 + KNxN0 + KNxN1 (Auto or Allograft)	45.7 (34.8-55.8)	99.8 (99.5-99.9)	89.6 (77.3-96.5)	97.8 (97.1-98.3)	48	43	2299	2248
7	St6 + KNxJ (Fracture surgery)	81.9 (72.6-89.1)	97.3 (96.5-97.9)	55.8 (47.1-64.2)	99.2 (98.8-99.6)	138	77	2209	2192
8	St7 + KNxB or KNxC (Primary or secondary prosthetic replacement)	92.6 (85.3-97.0)	93.2 (92.1-94.2)	36.2 (30.2-42.7)	99.7 (99.3-99.9)	240	87	2107	2100
9	St8 + KNxU49 (Removal og internal fixation device)	96.8 (91.0-99.3)	54.5 (52.4-56.6)	8.2 (6.6-9.9)	99.8 (99.3-99.9)	1116	91	1231	1228

A minimum of 42 reoperation days. Sensitivity, specificity, positive predictive value (PPV), and negative predictive value (NPV) for each step of the algorithm identifying nonunion. TP: Test positive, TrP: True positives, TN: test negative, TrN: True negative. Values are % with (95% confidence interval).

Table 4: Data from the different steps of the nonunion algorithm

Step	Accuracy	Recall	Precision	False positive rate	Kappa	AUC
1	0.97	0.18	0.94	0.00	0.29	0.59
2	0.97	0.20	0.95	0.00	0.32	0.60
3	0.97	0.22	0.95	0.00	0.35	0.61
4	0.97	0.28	0.90	0.00	0.41	0.64
5	0.97	0.39	0.90	0.00	0.54	0.70
6	0.98	0.46	0.90	0.00	0.60	0.73
7	0.97	0.82	0.56	0.03	0.65	0.90
8	0.93	0.93	0.36	0.07	0.49	0.95
9	0.56	0.97	0.08	0.45	0.08	0.95

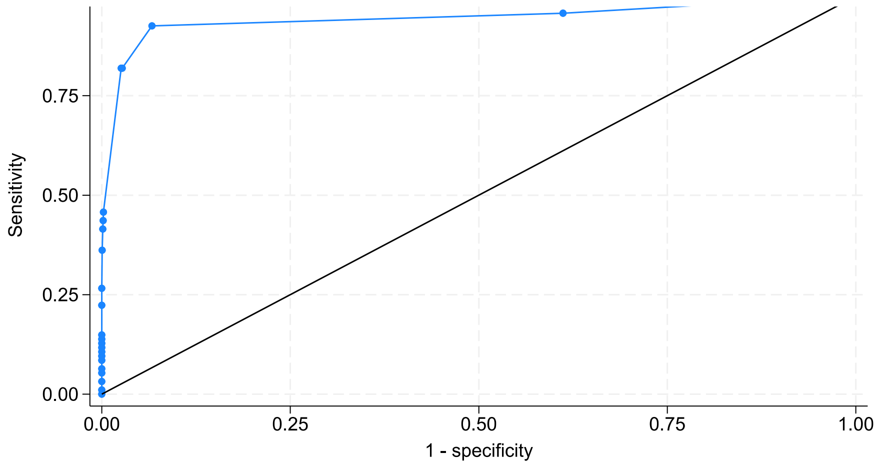

Figure 2: ROC-curve for nonunion algorithm

Table 5: Steps in the reosteosynthesis algorithm

Number of reosteosynthesis	195
Number of reoperations without reosteosynthesis	2152

Step	Codes	Sensitivity	Specificity	PPV	NPV	TP	TrP	TN	TrN
1	DT840-DT844 (Mechanical complications)	7.2 (4.0-11.8)	99.8 (99.5-99.9)	73.7 (48.8-90.9)	96.2 (95.3-96.9)	19	14	2328	2152
2	St1 + KNxJ (Fracture surgery)	87.2 (81.7-91.5)	97.4 (96.6-98.0)	74.9 (68.7-80.4)	98.8 (98.3-99.2)	227	170	2120	2095
3	St2 + KNxB (Primary prosthetic replacement)	89.7 (84.6-93.6)	97.3 (96.5-97.9)	75.1 (69.0-80.5)	99.1 (98.5-99.4)	233	175	2114	2094
4	St3 + KNxC (Secondary prosthetic replacement)	89.7 (84.6-93.6)	97.3 (96.5-97.9)	75.1 (69.0-80.5)	99.1 (98.5-99.4)	233	175	2114	2094

A maximum of 42 reoperation days. Sensitivity, specificity, positive predictive value (PPV), and negative predictive value (NPV) for each step of the algorithm identifying infectinos. TP: Test positive, TrP: True positives, TN: test negative, TrN: True negative. Values are % with (95% confidence interval).

Table 6: Data from the different steps of the reosteosynthesis algorithm

Step	Accuracy	Recall	Precision	False positive rate	Kappa	AUC
1	0.92	0.07	0.74	0.00	0.09	0.54
2	0.97	0.87	0.75	0.03	0.79	0.92
3	0.97	0.90	0.75	0.03	0.80	0.94
4	0.97	0.90	0.75	0.03	0.80	0.94

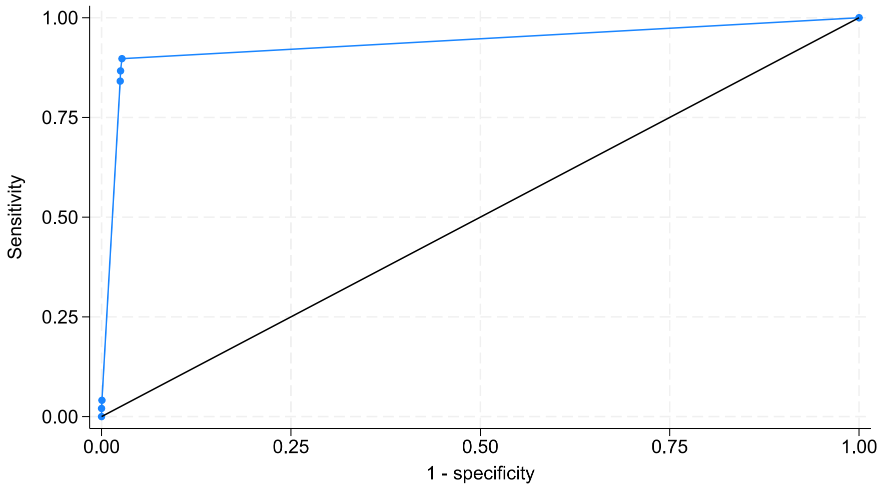

Figure 3: ROC-curve for reosteosynthesis algorithm

Table 7: Steps in the arthroplasty algorithm

Number of secondary arthroplasties	94
Number of reoperations without secondary arthroplasties	2253

Atep	Codes	Sensitivity	Specificity	PPV	NPV	TP	TrP	TN	TrN
1	KNxC (Secondary prosthetic replacement)	3.2 (0.7-9.0)	100.0 (99.8-100)	100 (29.2-100.0)	96.1 (95.2-96.9)	3	3	2344	2253
2	St1 + KNxB (Primary prosthetic replacement)	94.7 (88.0-98.3)	99.3 (98.9-99.6)	86.5 (77.3-91.7)	99.8 (99.5-99.9)	104	89	2243	2238

A minimum of 42 reoperation days. Sensitivity, specificity, positive predictive value (PPV), and negative predictive value (NPV) for each step of the algorithm identifying infectinos. TP: Test positive, TrP: True positives, TN: test negative, TrN: True negative. Values are % with (95% confidence interval).

Table 8: Data from the different steps of the arthroplasty algorithm

Step	Accuracy	Recall	Precision	False positive rate	Kappa	AUC
1	0.96	0.03	1.00	0.00	0.06	0.52
2	0.99	0.95	0.86	0.01	0.90	0.97

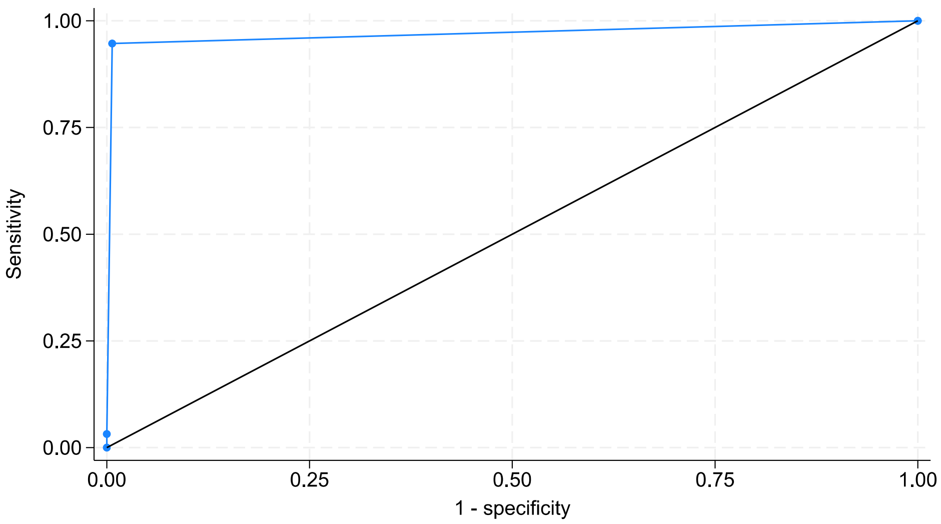

Figure 4: ROC-curve for arthroplasty algorithm
